# Interrogating Parkinson's disease LRRK2 kinase pathway activity by assessing Rab10 phosphorylation in human neutrophils

**DOI:** 10.1042/BCJ20170803

**Published:** 2018-01-02

**Authors:** Ying Fan, Andrew J.M. Howden, Adil R. Sarhan, Pawel Lis, Genta Ito, Terina N. Martinez, Kathrin Brockmann, Thomas Gasser, Dario R. Alessi, Esther M. Sammler

**Affiliations:** 1 MRC Protein Phosphorylation and Ubiquitylation Unit, School of Life Sciences, University of Dundee, Dundee DD1 5EH, U.K.; 2 Division of Cell Signalling and Immunology, School of Life Sciences, University of Dundee, Dundee, U.K.; 3 The Michael J. Fox Foundation for Parkinson's Research, Grand Central Station, PO Box 4777, New York, NY 10163, U.S.A.; 4 Department of Neurodegeneration, Hertie Institute for Clinical Brain Research, University of Tübingen, Tübingen 72076, Germany; 5 German Research Center for Neurodegenerative Diseases (DZNE), University of Tübingen, Tübingen, Germany; 6 Department of Neurology, School of Medicine, Ninewells Hospital, Ninewells Drive, Dundee DD1 9SY, U.K.

**Keywords:** biomarkers, diagnostics, leucine-rich repeat kinase, neutrophils, Parkinson's disease, Rab10

## Abstract

There is compelling evidence for the role of the leucine-rich repeat kinase 2 (LRRK2) and in particular its kinase function in Parkinson's disease. Orally bioavailable, brain penetrant and potent LRRK2 kinase inhibitors are in the later stages of clinical development. Here, we describe a facile and robust assay to quantify LRRK2 kinase pathway activity by measuring LRRK2-mediated phosphorylation of Rab10 in human peripheral blood neutrophils. We use the selective MJFF-pRab10 monoclonal antibody recognising the Rab10 Thr73 phospho-epitope that is phosphorylated by LRRK2. We highlight the feasibility and practicability of using our assay in the clinical setting by studying a few patients with G2019S LRRK2 associated and sporadic Parkinson's as well as healthy controls. We suggest that peripheral blood neutrophils are a valuable resource for LRRK2 research and should be considered for inclusion in Parkinson's bio-repository collections as they are abundant, homogenous and express relatively high levels of LRRK2 as well as Rab10. In contrast, the widely used peripheral blood mononuclear cells are heterogeneous and only a minority of cells (monocytes and contaminating neutrophils) express LRRK2. While our LRRK2 kinase pathway assay could assist in patient stratification based on LRRK2 kinase activity, we envision that it may find greater utility in pharmacodynamic and target engagement studies in future LRRK2 inhibitor trials.

## Introduction

Parkinson's disease is a common and complex neurodegenerative disorder affecting ∼1% of people over the age of 65 [1]. As with other neurodegenerative diseases, the greatest unmet need is the development of biomarkers and disease-modifying therapies. The risk for developing Parkinson's is multifactorial and results from an interplay between advancing age, environmental and genetic factors. The attributable risk of genetic factors ranges from highly penetrant mutations in ∼20 genes that cause rare, monogenetic forms of Parkinson's (all together accounting for less than 5–10% of all Parkinson's cases) to common variants with a mild-to-moderate effect size [2–4]. The leucine-rich repeat kinase 2 (LRRK2) gene is one of the main genetic contributors and was first discovered to be associated with Parkinson's in 2004 [5,6]. It is a large (2527 residues, 286 kDa) multi-domain protein including a ROC/COR GTPase and kinase catalytic domains [5,6]. LRRK2 not only constitutes a pleomorphic risk factor for developing Parkinson's, but also links familial and sporadic forms of the disease [7]. The frequency of LRRK2 mutations in autosomal dominant familial Parkinson's has been estimated to be ∼4% [8,9]. The G2019S LRRK2 mutation is particularly common and varies widely across populations — it is found in 5–10% of familial cases in Northern Europe and the U.S.A., but with much higher frequency, for example, in Portuguese patients (10%), Ashkenazi Jews (23%) and North African Berbers (40%) [10]. Interestingly, the G2019S LRRK2 mutation is also found in 1–2% of sporadic Parkinson's cases [10]. Its penetrance is incomplete and age-dependent [9]. In addition, there are common protein-coding and non-protein-coding variants at the LRRK2 locus that moderately increase the risk for developing Parkinson's [11].

Mounting evidence indicates that all pathogenic mutations, including G2019S, exert their effects by increasing LRRK2 kinase activity. As such there is considerable interest in targeting LRRK2 for the prevention and possibly treatment of Parkinson's [12]. Accordingly, pharmaceutical companies have generated highly selective, orally bioavailable and brain penetrant LRRK2 inhibitors that are in the later stages of clinical development [13].

Recent work has defined a subset of Rab GTPase proteins as the first validated physiological substrates of LRRK2 [14]. LRRK2 directly phosphorylates a conserved Thr/Ser residue residing at the centre of the effector-binding switch-II motif of many Rab proteins, including Rab10 (Thr73). All LRRK2 pathogenic mutations tested, including G2019S, increase the phosphorylation of Rab10 in cells (HEK293 and mouse embryonic fibroblasts) as well as mouse tissues (brain, spleen, lung and kidney) [14,15]. Specific phosphorylation of endogenous Rab10 has until now been assessed by either mass spectrometry analysis [14,16,17] or by employing the Phos-tag reagent which retards the electrophoretic mobility of LRRK2-phosphorylated Rab proteins [15]. As described in the accompanying paper, we have recently developed a highly sensitive rabbit monoclonal phospho-antibody (termed MJFF-pRab10) that detects Rab10 phosphorylated at Thr73 by LRRK2 [18]. Importantly, this antibody is highly specific and does not detect any of the other 13 Rab proteins known to be phosphorylated by LRRK2, a conclusion confirmed by using Rab10 knockout A549 cells [18].

LRRK2 is also constitutively phosphorylated at a cluster of serine residues that reside in a non-catalytic region between the Ankyrin domain and the leucine-rich repeat region (Ser910, Ser935, Ser955 and Ser973), and play a role in regulating 14-3-3 binding as well as cytosolic localisation [19,20]. These sites have received a lot of attention as they are controlled by LRRK2 kinase activity, and therefore become dephosphorylated in response to diverse LRRK2 inhibitors [21,22]. Thus, monitoring the dephosphorylation of these residues, especially Ser935, has become the principal pharmacodynamic marker to assess the *in vivo* efficacy of LRRK2 inhibitors in cell line and animal models [23]. However, a major drawback of Ser935 phosphorylation is that it does not correlate with intrinsic cellular LRRK2 kinase activity. For example, knock-in pathogenic mutations such as G2019S that increase LRRK2 kinase activity and Rab10 phosphorylation ∼2-fold have no effect on phosphorylation of Ser935 and other nearby phosphosites [14,15]. Moreover, Ser935 is still phosphorylated in kinase-inactive LRRK2, whereas Ser935 phosphorylation is strongly reduced in pathogenic mutations located in the ROC/COR GTPase domain that activate LRRK2 kinase activity to a greater extent than the G2019S mutation [15,19,24]. It is also not understood how phosphorylation of Ser935 and its close-by sites is controlled, and what the upstream kinase or kinases are that phosphorylate(s) these residues. Finally, it is also possible to assess LRRK2 activity by monitoring autophosphorylation of Ser1292, and phosphorylation of this site correlates well with LRRK2 kinase activity [25]. However, the available phospho-specific antibodies are insufficiently sensitive and/or phosphorylation stoichiometry is too low, to reliably utilise Ser1292 phosphorylation as a readout for endogenous LRRK2 kinase activity by immunoblot analysis of whole cell extracts.

In the present study, we explore the feasibility of assessing endogenous LRRK2 kinase activity in peripheral blood cells by monitoring LRRK2-mediated Rab10 phosphorylation, employing the newly developed MJFF-pRab10 phospho-specific rabbit monoclonal antibody [18]. We focus on human peripheral blood neutrophils as they constitute a homogenous cell population with high expression levels of both LRRK2 and Rab10. We argue that neutrophils are better suited than peripheral blood mononuclear cells (PBMCs) to study LRRK2 regulated Rab10 phosphorylation in human peripheral blood. Furthermore, we elaborate methods to quantitatively assess LRRK2-mediated Rab10 phosphorylation in human neutrophils including the study of a few clinical samples from LRRK2 G2019S associated and sporadic Parkinson's patients as well as controls. We anticipate that our LRRK2 kinase assay in neutrophils could have utility in future clinical trials to (1) assess LRRK2 pathway activity in Parkinson's patients, (2) identify patients displaying elevated LRRK2 kinase activity and (3) monitor pharmacokinetics and target engagement of administered LRRK2 inhibitors.

## Materials and methods

### Reagents

MLi-2 [26,27] and Phos-tag acrylamide [28] were synthesised at the University of Dundee. The PF-06447475 inhibitor was purchased from R&D systems (#5716), diisopropylfluorophosphate (DIFP) was from Sigma (Cat# D0879) and phenylmethane sulfonyl fluoride (PMSF) was from Sigma (Cat# 78830).

### Antibodies

MJFF-pRab10 rabbit monoclonal antibodies are described in the accompanying paper [18] and used at 1 µg/ml final concentration. This antibody will be made commercially available by the Michael J. Fox Foundation in the foreseeable future. To enable LI-COR multiplexing of the MJFF-pRab10 rabbit monoclonal antibodies with a total Rab10 antibody, we commissioned Nanotool Antibodies (http://www.nanotools.de/) to generate a mouse monoclonal antibody raised against human recombinant Rab10 (sequence 100% identical with mouse). The resultant antibody, termed MJFF-total Rab10, was highly selective and recognised only a single major band in wild type but not in previously described Rab10 knockout A549 cells [15] (Supplementary Figure S1). The MJFF-total Rab10 mouse monoclonal antibody was similarly sensitive and clean as the rabbit anti-Rab10 antibody from Cell Signaling Technology (#8127) used at a 1 : 1000 dilution. The selectivity of this antibody has previously been demonstrated by employing Rab10 knockout A549 cells [15] (Supplementary Figure S1). The MJFF-total Rab10 antibody will be made commercially available by the Michael J. Fox Foundation in the future. Total LRRK2 rabbit monoclonal antibody was raised against LRRK2 residues 100–500 (UDD3) and pS935-LRRK2 (UDD2) [24,29]. These antibodies were purified at the University of Dundee and used at 1 µg/ml final concentration. Mouse anti-LRRK2 C-terminus antibody was from Antibodies Incorporated (#75-253) and used at 1 : 1000 dilution. Anti-glyceraldehyde-3-phosphate dehydrogenase (GAPDH) antibody was from Santa Cruz Biotechnology (sc-32233) and used at 1 : 2000 dilution. Horseradish peroxidase-conjugated anti-mouse (#31450) and -rabbit (#31460) secondary antibodies were from Thermo Fisher Scientific and used at 1 : 5000 dilution. Goat anti-mouse IRDye 800CW (#926-32210) and IRDye 680LT (#926-68020) and goat anti-rabbit IRDye 800CW ((#926-32211) secondary antibodies were from LI-COR.

### Study participants and blood sample collection

For setting up and validating the LRRK2-mediated Rab10 assay, we recruited volunteers from within the School of Life Sciences at the University of Dundee who kindly donated blood for the present study. Specifically, the data shown in Figures 2 and 6–10 and Supplementary Figures S2 and S3 are derived from healthy volunteers from Dundee.

For the clinical part of our study, blood was collected from a total of 26 subjects that were associated with the Neurology Department at the University of Tuebingen in Germany: seven patients with sporadic Parkinson's and five patients with G2019S LRRK2 associated Parkinson's as well as 1 G2019S LRRK2 non-manifesting carrier and 13 healthy controls. Most of the control subjects were enrolled from the well-characterized TREND (Tubingen evaluation of Risk factors for early detection of neurodegeneration) study as well as some others from within staff from the Neurology Department and two healthy family members (one unrelated healthy spouse and one daughter, who had tested negative for the G2019S LRRK2 mutation). Demographics, such as gender, age, disease duration and ethnicity, were collected. Diagnosis of Parkinson's was defined according to the UK Brain Bank criteria with the exception that a positive family history for Parkinson's was not considered an exclusion criterion [30]. The severity of motor symptoms and the presence of motor complications were assessed using part III and IV of the Movement disorder society — Unified Parkinson's disease rating scale (MDS-UPDRS-III and -IV) [31]. Cognitive function was tested with the Montreal Cognitive Assessment (MoCA) [32]. A cut off of <26 out of 30 points indicated cognitive impairment. Levodopa-equivalent daily dose (LEDD) was recorded as well as any additional non-oral therapies such as deep brain stimulation. Study participants' demographics and clinical information can be found in Supplementary Tables S1 and S2.

### Ethical approval and consent to participate

The study was approved by the respective local ethics committees of the University of Dundee in the United Kingdom and the University of Tuebingen in Germany. All participants gave written informed consent.

### Neutrophil isolation, characterisation, treatments and lysis

Neutrophils were isolated directly from human whole blood by immune-magnetic negative isolation using the EasySep Direct Human Neutrophil Isolation Kit (STEMCELL Technologies, Cat# 19666). Twenty millilitres of blood were transferred into a 50 ml falcon tube containing 200 µl of 100 mM EDTA in phosphate-buffered saline (PBS) to obtain a final concentration of EDTA at 1 mM, and tubes were gently mixed by inversion. One millilitre of ‘Isolation Cocktail’ from the neutrophil isolation kit was added to the whole blood (i.e. 50 µl/ml of blood). The ‘RapidSpheres’ magnetic beads from the isolation kit were resuspended by vortexing for 30 s and 1 ml of beads were added to the whole blood (i.e. 50 µl/ml of blood). The blood sample containing the magnetic beads was gently mixed by inversion and incubated at room temperature for 5 min. The blood sample was then diluted to a final volume of 50 ml with 1 mM EDTA in PBS and then gently mixed by inversion of the tube. The falcon tube was next placed into the Easy 50 EasySep Magnet (STEMCELL Technologies, Cat# 18002) without lid (to avoid subsequent agitation of tube) and incubated for 10 min at room temperature to remove non-neutrophil blood cells. The supernatant (∼40 ml) containing the enriched neutrophils was carefully pipetted into a new 50 ml falcon tube, avoiding disrupting the magnetic beads attached to the sides of the falcon tube that is in contact with the magnet as well as the red blood cells that were accumulated at the bottom of the tube (typically present in the remaining ∼10 ml). To further purify neutrophils, another 1 ml of resuspended RapidSpheres magnetic beads was added to the enriched neutrophils (i.e. 50 µl/ml of blood), the tube was gently mixed by tube inversion and incubated at room temperature for 5 min. The tube was then placed into the EasySep Magnet without lid and after 5 min incubation at room temperature, the supernatant containing highly purified neutrophils was carefully pipetted into a new 50 ml falcon tube, taking care to collect only the clear fraction. To ensure complete removal of magnetic beads from the cell mixture, the resulting neutrophils were placed for a final time in the magnet (without lid). After a 10 min incubation at room temperature, the pure neutrophil cell suspension was carefully pipetted into a new tube, ensuring that only the clear fraction was collected (which will be ∼30 ml at this stage). The resulting isolated neutrophils were diluted in PBS containing 1 mM EDTA to a final volume of 41 ml and then divided equally into two tubes (each containing 20 ml), one for LRRK2 inhibitor MLi-2 treatment and one for vehicle control DMSO treatment, and 1 ml of neutrophil suspension was kept for cell counting, viability and purity analysis using flow cytometer. The two 20 ml neutrophil preparations were then centrifuged at 335 ***g*** for 5 min (acceleration and deceleration is both 5 using a Beckman Coulter Allegra X-15R Centrifuge), and the supernatant was carefully poured away. The neutrophil cell pellets were resuspended with 10 ml room temperature RPMI 1640 media by gentle pipetting. At this stage, purified neutrophils were subjected to with or without MLi-2 treatment at concentrations and periods of time as indicated in each figure legend (typically 30 min). MLi-2 was dissolved in DMSO at a concentration of 1000-fold higher than the final concentrations added to neutrophils. An equivalent volume of DMSO was added to negative control samples. Following treatment, neutrophils were pelleted through centrifugation at 335 ***g*** for 5 min. Neutrophil pellets were then resuspended in 1 ml of RPMI 1640 media with or without original concentrations of MLi-2, transferred into an Eppendorf tube and centrifuged at 300 ***g*** for 3 min. The supernatant was carefully removed by pipetting, and neutrophil pellet was lysed with 100 µl of ice-cold lysis buffer containing 50 mM Tris–HCl, pH 7.5, 1% (v/v) Triton X-100, 1 mM EGTA, 1 mM sodium orthovanadate, 50 mM NaF, 0.1% (v/v) 2-mercaptoethanol, 10 mM 2-glycerophosphate, 5 mM sodium pyrophosphate, 0.1 µg/ml mycrocystin-LR (Enzo Life Sciences), 270 mM sucrose, 0.5 mM DIFP (Sigma, Cat# D0879) in addition to Complete EDTA-free protease inhibitor cocktail (Sigma–Aldrich, Cat# 11836170001). DIFP is highly toxic and must be prepared in a fume hood to a stock solution of 0.5 M in isopropanol. This stock solution of DIFP is stored at −80°C and stable for over 1 year. Dr Len Stephens from the Babraham Institute in Cambridge, U.K., recommended that DIFP was used instead of PMSF for neutrophil lysis buffer, in order to suppress the intrinsic serine protease activity that is known to be high in neutrophils [33] (D. Alessi personal communication). DIFP is added freshly to lysis buffer from the isopropanol solution in a fume hood just prior to lysing cells. Neutrophil cell lysates were clarified by centrifugation at 20 800 ***g*** for 15 min at 4°C. Supernatants were used for Bradford assay (Thermo Scientific) and snap-frozen and stored at −80°C.

The viability and purity of neutrophils after isolation using the ∼1 ml remaining sample was checked through flow cytometry analysis. Isolated cells were incubated with 2.5 µg of human FC block (BD Biosciences, Cat# 564220) in 100 µl FACS buffer for 10 min at room temperature. FACS buffer comprised of Dulbecco's PBS (Thermo Fisher Scientific, Cat# 14190094) + 1% foetal bovine serum (FBS) (Thermo Fisher Scientific, Cat# 10270106). After blocking, cells were stained with the granulocyte-specific cell surface marker CD66b using anti-human CD66b-FITC antibody (BD Biosciences, Cat# 555724, clone G10F5). Antibody was used at a final dilution of 1 : 5 according to the manufacturer's instructions. Cells were also stained with 4′,6-diamidino-2-phenylindole (DAPI, Thermo Fisher Scientific, Cat# D1306) at 2.5 µg/ml before analysis on a FACSVerse flow cytometer with FACSuite software (BD Biosciences). Data were analysed using FlowJo software (FlowJo, LLC).

### PBMC isolation, characterisation, treatments and lysis

PBMCs were purified from 15 ml human whole blood via density centrifugation using Ficoll-Paque PREMIUM (GE Healthcare, Cat# 17-5442-02). Blood was collected using BD Vacutainer sodium heparin (17 IU/ml) tubes, green closure 16 × 100 mm (BD, cat# 368480). Fifteen millilitres of Ficoll-Paque were added into the SepMate™ tube (STEMCELL, Cat# 85450, 50 ml capacity) using a syringe through the central hole of the SepMate™ insert to completely fill the bottom cavity. Whole blood (15 ml) was diluted with an equal amount (15 ml) of PBS containing 2% (by vol) FBS before being added into the top part of the SepMate™ tube. After centrifugation at 1200 ***g*** for 10 min at room temperature (with the brake ON), the top plasma transparent layer was discarded and the remainder cloudy layer of sample above the insert disk that contains the PBMCs was collected and transferred to a new 15 ml falcon tube. The sample was then diluted with PBS containing 2% (by vol) FBS to a final volume of 15 ml, and the cells were gently mixed by tube inversion. PBMCs were pelleted by centrifugation at 1000 ***g*** for 2 min. The supernatant was discarded by pouring and the PBMC pellet was resuspended in 15 ml of PBS containing 2% FBS (by vol) for a second round of washing. After centrifugation at 1000 ***g*** for 2 min, the PBMC pellet was then resuspended in 10.5 ml PBS containing 2% FBS. The PBMC suspension was aliquoted into two tubes of 5 ml each for with or without MLi-2 inhibitor treatment, and the remaining ∼0.5 ml of PBMCs used for, viability, and purity analysis by flow cytometry analysis. At this stage, purified PBMCs were subjected to with or without 200 nM MLi-2 treatment for 30 min. MLi-2 was dissolved in DMSO at a concentration of 200 µM (1000-fold higher than the final concentration) and 5 µl was added to the +MLi-2 sample and an equivalent volume of DMSO (5 µl) also added to the −MLi-2 sample. Following 30 min incubation at room temperature, the supernatant was discarded by pouring. PBMC pellets are then resuspended in 0.5 ml of PBS containing 2% FBS with or without 200 nM MLi-2, transferred into an Eppendorf tube and centrifuged at 300 ***g*** for 3 min. The supernatant was carefully removed by pipetting and PBMC pellet was lysed with 200 µl of ice-cold lysis buffer containing 50 mM Tris–HCl, pH 7.5, 1% (v/v) Triton X-100, 1 mM EGTA, 1 mM sodium orthovanadate, 50 mM NaF, 0.1% (v/v) 2-mercaptoethanol, 10 mM 2-glycerophosphate, 5 mM sodium pyrophosphate, 0.1 µg/ml mycrocystin-LR (Enzo Life Sciences), 270 mM sucrose, 0.5 mM DIFP (Sigma, Cat# D0879) in addition to Complete EDTA-free protease inhibitor cocktail (Sigma–Aldrich Cat # 11836170001). DIFP is highly toxic and must be prepared in a fume hood to a stock solution of 0.5 M in isopropanol. This stock solution of DIFP is stored at −80°C and stable for over 1 year. DIFP is added freshly to lysis buffer from the isopropanol solution in a fume hood just prior to lysing cells. PBMC cell lysates were clarified by centrifugation at 20 800 ***g*** for 15 min at 4°C. Supernatants were used for the Bradford assay (Thermo Scientific) and snap-frozen and stored at −80°C.

For viability and cell population determination via flow cytometry analysis, PBMCs were FC-blocked as described above for neutrophils, before staining with the following cell surface markers: CD19 APC (B-cell marker, BD Biosciences, Cat# 561 742, clone HIB19) at 1 : 5 dilution, CD14 V500 (monocyte marker, BD Biosciences, Cat# 561392, clone M5E2) at 1 : 20 dilution, CD3 PE (T-cell marker, BD Biosciences, Cat# 561808, clone UCHT1) at 1 : 5 dilution and CD66b FITC (granulocyte marker, BD Biosciences, Cat# 555724, clone G10F5) at 1 : 5 dilution. Cells were also stained with DAPI (Thermo Fisher Scientific, Cat# D1306) at 2.5 µg/ml before analysis on a FACS LSR Fortessa flow cytometer with DIVA software (BD Biosciences). Data were analysed using FlowJo software (FlowJo, LLC).

### Quantitative immunoblot analysis

Cell lysates were mixed with 4× SDS–PAGE loading buffer [250 mM Tris–HCl, pH 6.8, 8% (w/v) SDS, 40% (v/v) glycerol, 0.02% (w/v) Bromophenol Blue and 4% (v/v) 2-mercaptoethanol] to a final total protein concentration of 1 µg/µl and heated at 70°C for 10 min. Ten microgram of samples were loaded onto NuPAGE 4–12% Bis–Tris Midi Gel (Thermo Fisher Scientific, Cat# WG1403BOX) and electrophoresed at 130 V for 2 h with the NuPAGE MOPS SDS running buffer (Thermo Fisher Scientific, Cat# NP0001-02). At the end of electrophoresis, proteins were electrophoretically transferred onto the nitrocellulose membrane (GE Healthcare, Amersham Protran Supported 0.45 µm NC) at 100 V for 90 min on ice in the transfer buffer (48 mM Tris–HCl and 39 mM glycine). Transferred membrane was blocked with 5% (w/v) skim milk powder dissolved in TBS-T [20 mM Tris–HCl, pH 7.5, 150 mM NaCl and 0.1% (v/v) Tween 20] at room temperature for 1 h. The membrane was then cropped into three pieces, namely the ‘top piece’ (from the top of the membrane to 75 kDa), the ‘middle piece’ (between 75 and 30 kDa) and the ‘bottom piece’ (from 30 kDa to the bottom of the membrane). The top piece was incubated with rabbit anti-LRRK2 pS935 UDD2 antibody multiplexed with mouse anti-LRRK2 C-terminus total antibody diluted in 5% (w/v) skim milk powder in TBS-T to a final concentration of 1 µg/ml for each of the antibody. The middle piece was incubated with mouse anti-GAPDH antibody diluted in 5% (w/v) skim milk powder in TBS-T to a final concentration of 50 ng/ml. The bottom pieces were incubated with rabbit MJFF-pRAB10^clone-1^ monoclonal antibody multiplexed with mouse MJFF-total Rab10^clone-1^ monoclonal antibody diluted in 2% (w/v) bovine serum albumin in TBS-T to a final concentration of 0.5 µg/ml for each of the antibody. All blots were incubated in primary antibody overnight at 4°C. Prior to secondary antibody incubation, membranes were washed three times with TBS-T for 10 min each. The top and bottom pieces were incubated with goat anti-mouse IRDye 680LT (#926-68020) secondary antibody multiplexed with goat anti-rabbit IRDye 800CW ((#926-32211) secondary antibody diluted in TBS-T (1 : 10 000 dilution) for 1 h at room temperature. The middle piece was incubated with goat anti-mouse IRDye 800CW (#926-32210) secondary antibody diluted in TBS-T (1 : 10 000 dilution) at room temperature for 1 h. Membranes were washed with TBS-T for three times with a 10 min incubation for each wash. Protein bands were acquired via near infrared fluorescent detection using the Odyssey CLx imaging system and quantified using the Image Studio software.

### Phos-tag polyacrylamide gel electrophoresis

Phos-tag acrylamide was stored at 5 mM aqueous solution (3.43 mg of compound in 1 ml of solution) at −80°C in black tubes that block out light as Phos-tag acrylamide is light-sensitive. HPLC analysis of stock Phos-tag acrylamide was undertaken every 4–5 weeks to ensure stock had not started to polymerise. Phos-tag analysis including sample preparation was undertaken as described recently [15]. Briefly, Phos-tag SDS–PAGE samples were supplemented with MnCl_2_ to a final concentration of 10 mM before loading gels. Gels for Phos-tag SDS–PAGE consisted of a stacking gel [4% (w/v) acrylamide, 125 mM Tris–HCl, pH 6.8, 0.1% (w/v) SDS, 0.2% (v/v) *N*,*N*,*N*′,*N*′-tetramethylethylenediamine (TEMED) and 0.08% (w/v) ammonium persulfate (APS)] and a separating gel [12% (w/v) acrylamide, 375 mM Tris/HCl, pH 8.8, 0.1% (w/v) SDS, 75 µM Phos-tag acrylamide, 150 µM MnCl2, 0.1% (v/v) TEMED and 0.05% (w/v) APS]. After centrifugation at 20 800 ***g*** for 1 min, 10 µg of samples were loaded and electrophoresed at 70 V for the stacking part and at 120 V for the separating part with the running buffer [25 mM Tris–HCl, 192 mM glycine and 0.1% (w/v) SDS]. For immunoblot analysis, gels were washed for 10 min in the transfer buffer [48 mM Tris–HCl, 39 mM glycine and 20% (v/v) methanol] containing 10 mM EDTA and 0.05% (w/v) SDS three times, followed by one wash in the transfer buffer containing 0.05% SDS for 10 min. Proteins were electrophoretically transferred onto nitrocellulose membranes (Amersham Protran 0.45 µm NC; GE Healthcare) at 100 V for 180 min on ice in the transfer buffer (48 mM Tris–HCl and 39 mM glycine). Transferred membranes were blocked with 5% (w/v) skim milk powder dissolved in TBS-T [20 mM Tris–HCl, pH 7.5, 150 mM NaCl and 0.1% (v/v) Tween 20] at room temperature for 1 h. Membranes were then incubated with primary antibodies diluted in 5% (w/v) skim milk powder or 2% (w/v) bovine serum albumin in TBS-T overnight at 4°C. After washing membranes in TBS-T, membranes were incubated with secondary antibody (horseradish peroxidase-labelled secondary antibodies diluted in 5% skim milk powder in TBS-T) for Phos-tag immunoblot at room temperature for 1 h. After washing membranes in TBS-T, protein bands were detected by exposing films [Amersham Hyperfilm ECL (GE Healthcare)] to the membranes using an ECL solution [SuperSignal West Dura Extended Duration (Thermo Fisher Scientific)].

## Results

### Neutrophils are a homogenous peripheral blood cell population that express high levels of LRRK2 and Rab10

**Figure 1.**
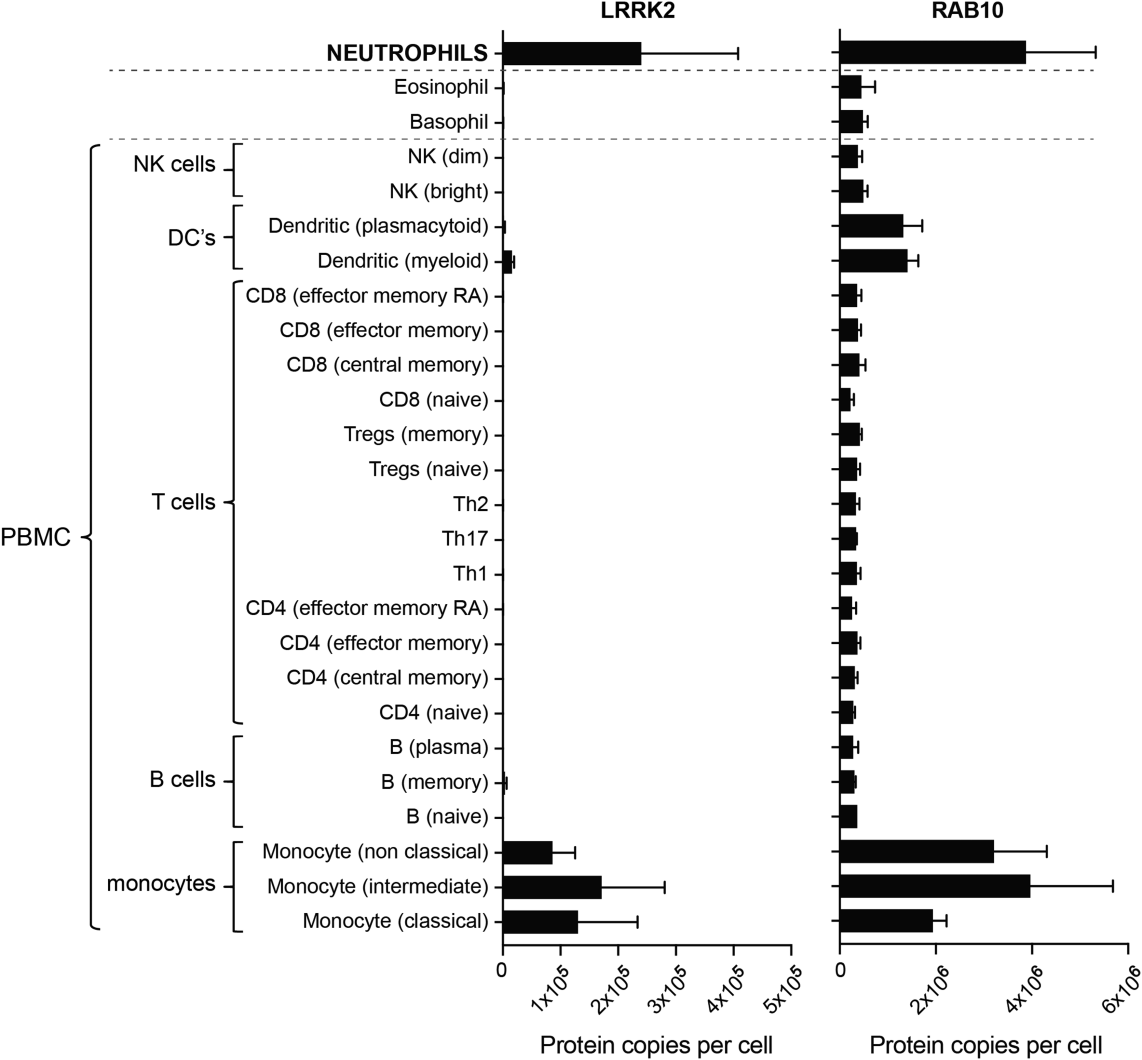
The abundance of LRRK2 and RAB10 proteins in immune cells isolated from human blood using data available from the immprot database (http://www.immprot.org**)** [34]. The study utilised pure populations of immune cells isolated from human blood using fluorescence-activated cell sorting, and whole cell proteomics data were generated. Data were analysed using the histone ruler to estimate protein copy numbers per cell. The graphs show the number of protein copies per cell for LRRK2 and RAB10 in a range of peripheral blood immune cells including subsets of T cells, B cells, monocytes, NK cells, dendritic cells and the granulocytes neutrophils, basophils and eosinophils.

To gain insights into the expression of the LRRK2 and Rab10 proteins in human peripheral blood cells, we interrogated the IMMPROT proteomic database (http://www.immprot.org/) [34]. This suggests that LRRK2 is highly expressed in neutrophils (∼2 × 10^5^ copies per cells) as well as monocytes (∼1 × 10^5^ copies per cells), but is virtually undetectable in B and T lymphocytes as well as natural killer and dendritic cells that constitute most of the PBMCs (Figure 1). Rab10 is also highly expressed in neutrophils and monocytes (2–4 × 10^6^ copies per cells) and is up to 10-fold less abundant in all other PBMCs (2–4 × 10^5^ copies per cell) (Figure 1). Blood cell-type frequencies vary from donor to donor; however, neutrophils are the most abundant white blood cell (37–80% of the white blood cells), while PBMCs are a heterogeneous cell population and are generally less abundant (10–62% of the white blood cells) [35].

### Analysis of LRRK2-mediated Rab10 phosphorylation in human neutrophils by quantitative immunoblot analysis

**Figure 2.**
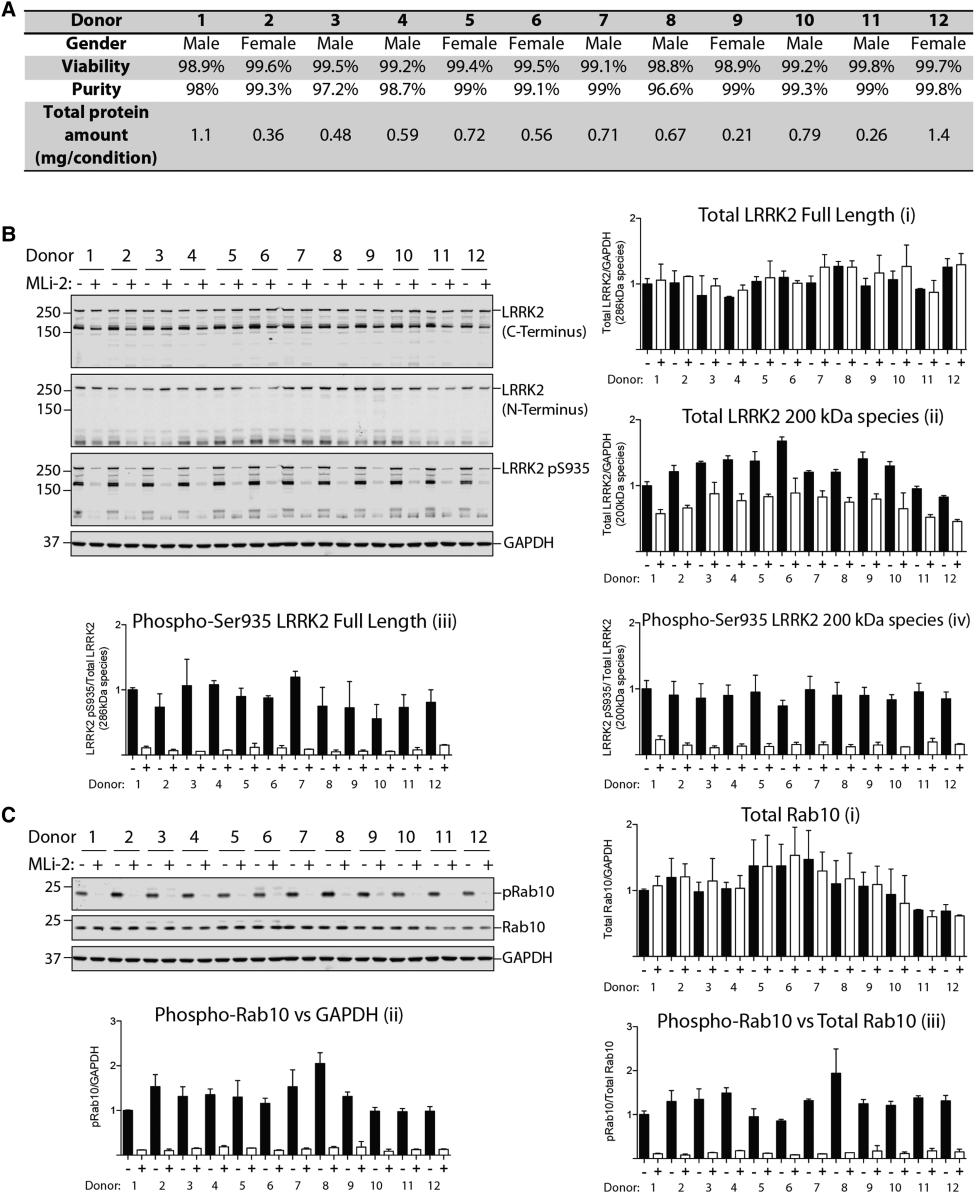
Monitoring LRRK2-mediated Ser935 and Rab10 phosphorylation in human neutrophils. Neutrophils were isolated from 12 healthy donors and treated with or without 100 nM MLi-2 for 30 min. (**A**) Viability (DAPI) and purity (CD66b) of neutrophils isolated from whole blood of 12 healthy volunteers were assessed via flow cytometer analysis. (**B** and **C**) Neutrophils were then lysed and 10 µg of whole cell extract subjected to quantitative immunoblot analysis with the indicated antibodies (all at 1 µg/ml antibody) and the membranes were developed using the Odyssey CLx scan Western Blot imaging system. Similar results were obtained in two independent experiments. Quantitation of immunoblots was undertaken by analysing LRRK2 (full length)/GAPDH ratio (**Bi**), total LRRK2 (170 kDa species)/GAPDH ratio (**Bii**), phospho-Ser935 LRRK2 (full length)/total LRRK2 (full length) ratio (**Biii**) and phospho-Ser935 LRRK2 (170 kDa species)/total LRRK2 (170 kDa species) ratio (**Biv**), total Rab10/GAPDH ratio (**Ci**), phospho-Thr73 Rab10/GAPDH ratio (**Cii**) and phospho-Thr73 Rab10/total Rab10 ratio (**Ciii**).

To our knowledge, the LRRK2 pathway activity has not been assessed in human neutrophils before. We therefore isolated neutrophils from peripheral blood from 12 healthy volunteers exploiting an immunomagnetic negative isolation approach, in which all non-neutrophils are targeted for removal with antibody complexes recognizing unwanted cells, including red blood cells and platelets, leaving only neutrophils in the supernatant. Flow cytometry analysis with the CD66b− Fluorescein isothiocyanate neutrophil marker revealed that the purity of neutrophils isolated from each volunteer was 97–99% and viability of cells was ∼99% assessed with DAPI staining (Figure 2A and Supplementary Figure S2). From 20 ml of blood, we obtained 0.2–1.4 mg of total protein from each donor (Figure 2A), sufficient for a significant number of immunoblot analysis that requires only 10 µg per gel lane due to the high-quality antibodies that were deployed for the assay.

To ensure that observed LRRK2 Ser935 and Rab10 phosphorylation was mediated by LRRK2, we incubated each batch of neutrophils with or without 100 nM MLi-2, a very selective and potent LRRK2 inhibitor [26,27]. Neutrophils were lysed in a buffer containing the protease inhibitor DIFP in order to suppress intrinsic serine protease activity that is known to be high in these cells [33]. Immunoblot analysis with an LRRK2 total antibody recognising the C-terminal domain revealed two species migrating at ∼286 kDa (corresponding to full-length LRRK2) and ∼170 kDa (Figure 2B). The ∼170 kDa LRRK2 species was 2- to 3-fold more abundant than the full-length LRRK2 and is likely to correspond to an N-terminally truncated form, as it is not recognised by an antibody raised against the N-terminal domain of LRRK2 (Figure 2B). The levels of the full-length LRRK2 were very similar in all samples (Figure 2Bi), but levels of the ∼170 kDa species varied more (Figure 2Bii). Furthermore, we also noticed that treatment with MLi-2 induced a moderate reduction in the amount of the ∼170 kDa species (Figure 2Bii) without affecting levels of the full-length form (Figure 2Bi). Immunoblotting with the LRRK2 Ser935 phospho-specific antibody revealed that the full-length and ∼170 kDa species of LRRK2 were phosphorylated at Ser935 and both became dephosphorylated following MLi-2 treatment (Figure 2Biii,iv). Quantitative immunoblot analysis revealed that both the full-length and the ∼170 kDa species of LRRK2 are similarly phosphorylated at Ser935 in all 12 neutrophil samples, with less than 1.5-fold variance between the samples (Figure 2Biii,iv).

Immunoblotting using the MJFF-pRab10 monoclonal antibody revealed a robust signal in all neutrophil samples, which was markedly suppressed by MLi-2 LRRK2 inhibitor treatment (Figure 2C). Rab10 protein expression was also similar in all 12 neutrophil samples with less than 2-fold difference observed (Figure 2Ci). We quantified Rab10 phosphorylation by either normalising against GAPDH (Figure 2Cii) or total Rab10 (Figure 2Ciii), and both revealed a similar relative pattern of phosphorylation between samples. The level of Rab10 phosphorylation varied ∼2-fold among samples, greater than that observed with Ser935 phosphorylation (Figure 2Biv,Ciii). For example, donor 6 (low Rab10 phosphorylation) and donor 8 (high Rab10 phosphorylation) have an ∼2-fold difference in Rab10 phosphorylation (Figure 2Ciii), while displaying similar levels of Ser935 phosphorylation (Figure 2Biii,iv).

### LRRK2-mediated Rab10 phosphorylation in neutrophils from control and Parkinson's patients with and without the LRRK2 G2019S mutation

We next explored the feasibility of analysing LRRK2 pathway activity in neutrophils isolated from a small number of Parkinson's patients with and without the G2019S LRRK2 mutation. The purpose of the present study was to test the practicability of assessing LRRK2-mediated Rab10 phosphorylation in a patient cohort in a clinical setting and not to address whether Rab10 phosphorylation is elevated in patients with Parkinson's or G2019S carriers. To answer this question, further studies with much larger numbers of patients and controls will be required.

**Figure 3.**
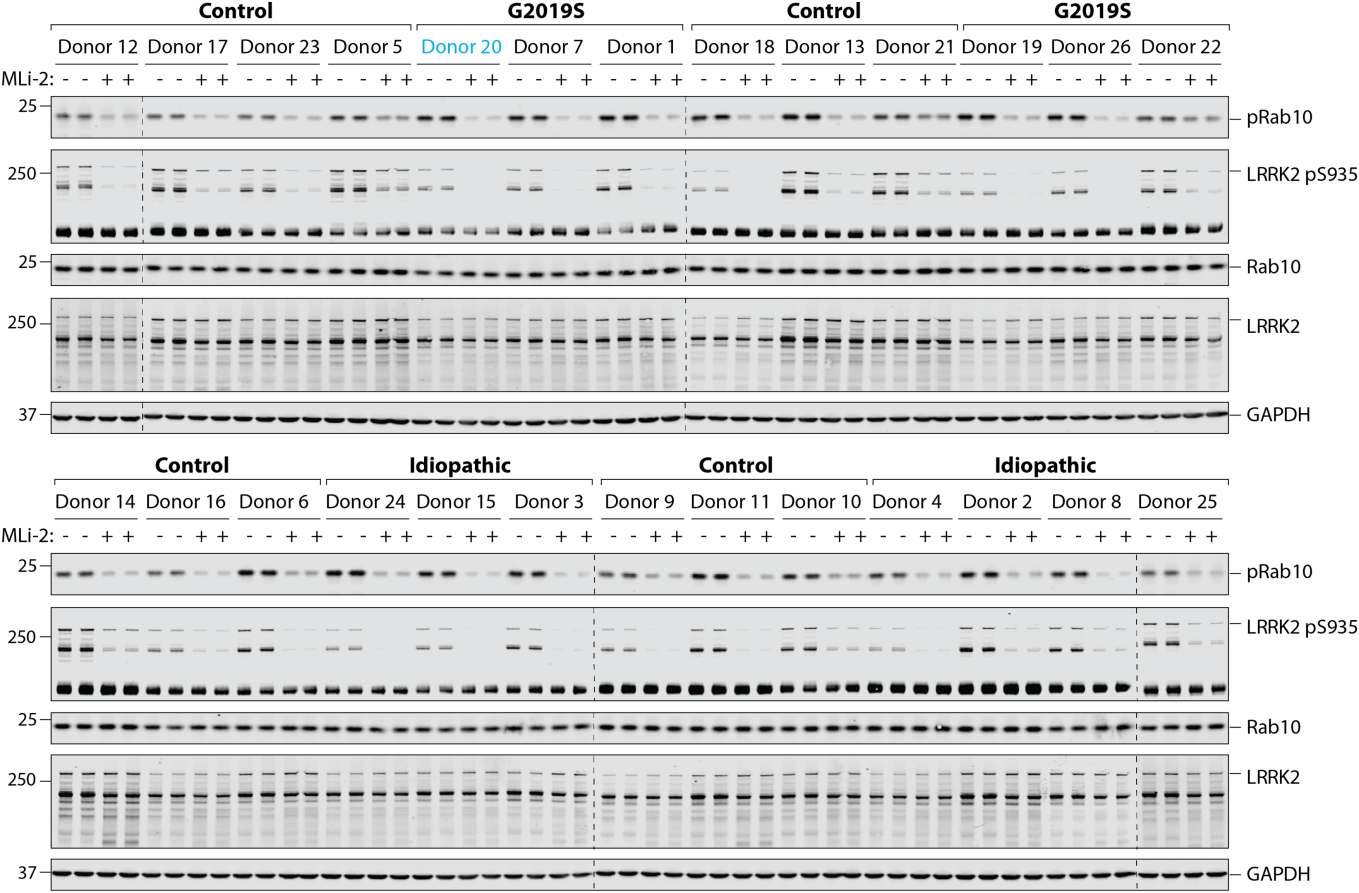
Analysing LRRK2-mediated Rab10 phosphorylation in neutrophils from control and Parkinson's patients. Neutrophils were isolated from 13 healthy controls, 7 sporadic Parkinson's disease patients and 6 individuals with a heterozygous G2019S LRRK2 mutation — 5 with Parkinson's disease and 1 carrier (Donor 20, highlighted in blue). Cells were treated with or without 100 nM MLi-2 for 30 min. Cells were then lysed and 10 µg of whole cell extract subjected to quantitative immunoblot analysis with the indicated antibodies (all at 1 µg/ml antibody), and the membranes developed using the Odyssey CLx scan Western Blot imaging system. Similar results were obtained in three independent immunoblot experiments of the same extracts.

**Figure 4.**
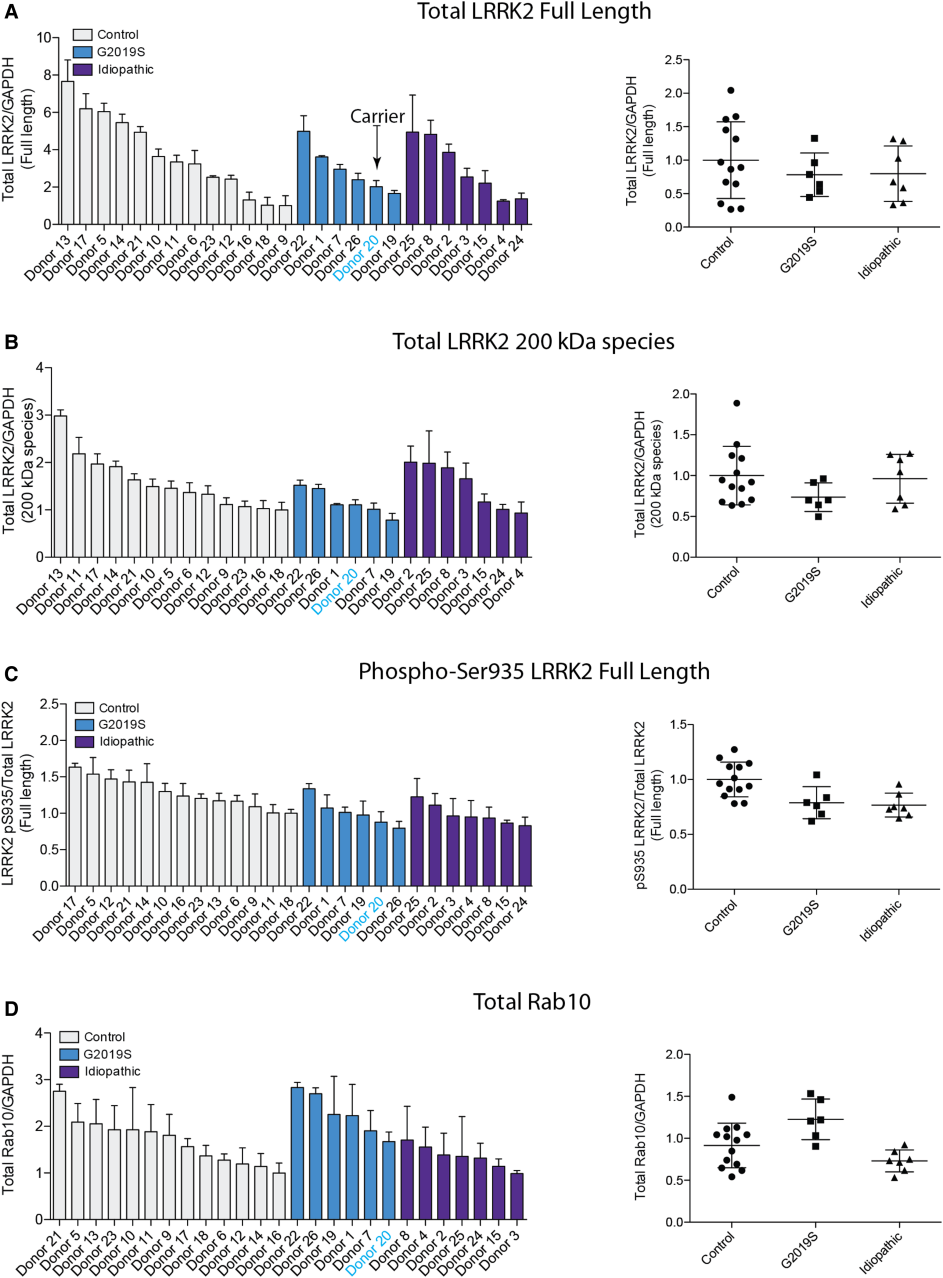
Quantitation of LRRK2, LRRK2 Ser935 phosphorylation and Rab10 in neutrophils from control and Parkinson's patients. The immunoblots from Figure 3 were quantified for full-length LRRK2/GAPDH ratio (**A**), ∼170 kDa species of LRRK2/GAPDH ratio (**B**), phospho-Ser935 LRRK2 (full length)/total LRRK2 (full length) ratio (**C**) and total Rab10/GAPDH ratio (**D**).

**Figure 5.**
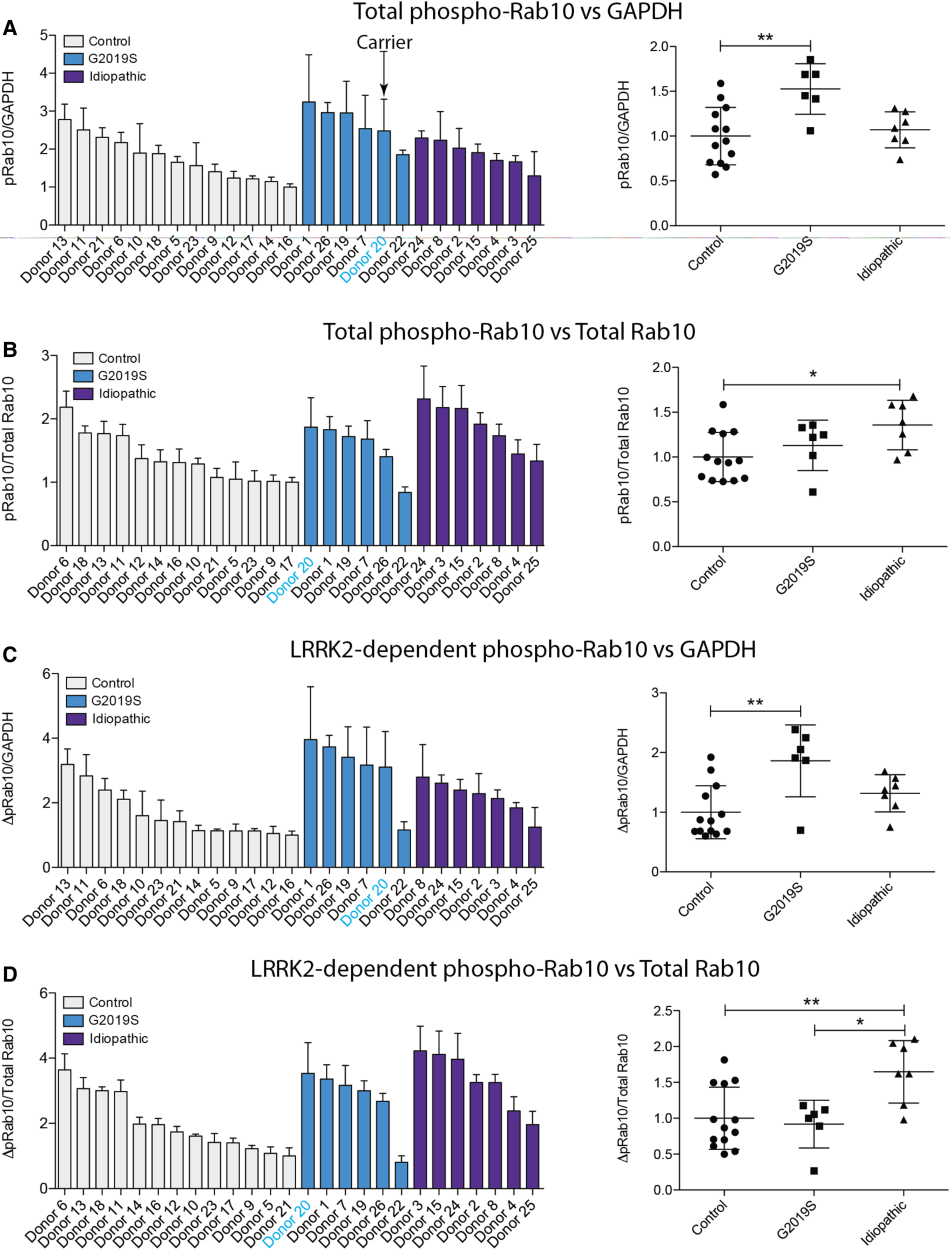
Quantitation of LRRK2-mediated Rab10 phosphorylation in neutrophils from control and Parkinson's patients. The immunoblots from Figure 3 were quantified for phospho-Thr73 Rab10/GAPDH ratio (**A**), phospho-Thr73 Rab10/total Rab10 ratio (**B**), LRRK2-dependent phospho-Thr73 Rab10/GAPDH ratio (**C**) and LRRK2-dependent phospho-Thr73 Rab10/total Rab10 ratio (**D**). Data were analysed by one-way ANOVA with Bonferroni's multiple comparisons test. Data presented as means ± SD; *P < 0.05, **P < 0.005.

Neutrophils were isolated from 13 healthy controls, 7 patients with sporadic Parkinson's and 6 individuals with a heterozygous G2019S LRRK2 mutation — 5 with Parkinson's and 1 non-manifesting carrier (Figures 3–5). Demographic and clinical data for each subject are presented in Supplementary Tables S1 and S2. As before, each participant's neutrophils were treated with or without 100 nM MLi-2 LRRK2 inhibitor for 30 min at room temperature before cell lysis. Samples were subjected to quantitative immunoblot analysis with total LRRK2, pSer935 LRRK2, total Rab10, MJFF-pRAB10 (pThr73) and a loading control GAPDH antibody. Levels of full-length LRRK2 varied by ∼8 fold among all the donors (Figure 4A), while levels of ∼170 kDa LRRK2 species varied ∼3-fold (Figure 4B). Ser935/total full-length LRRK2 varied ∼1.5-fold between donors (Figure 4C), while there was ∼3-fold variance between total Rab10 levels (Figure 4D) with no significant differences between healthy controls, sporadic Parkinson's disease and heterozygous G2019S LRRK2 mutation carriers for any of these analysis (Figure 4).

We quantified Rab10 phosphorylation in four different ways, by normalising pRab10 signal obtained with no MLi-2 treatment with either GAPDH (Figure 5A) or total Rab10 (Figure 5B), or by subtracting the phosphorylated Rab10 signal from samples treated plus MLi-2 from untreated samples and normalising with GAPDH (Figure 5C) or total Rab10 (Figure 5D). As observed with the healthy donor samples (Figure 2), there was greater variation in Rab10 phosphorylation between the individual participants in our study and a clear reduction in Rab10 phosphorylation upon LRRK2 inhibition (Figure 5). The data is broadly comparable for all four ways of quantifying Rab10 phosphorylation. For example, donor 22 (G2019S) and donor 25 (sporadic) consistently show the lowest level of Rab10 phosphorylation, whichever way the data are quantified (Figure 5). Similarly, donor 1 and donor 20 (both G2019S) as well as donor 8 and donor 24 (both sporadic) display among the highest levels of Rab10 phosphorylation in all ways that the data are analysed (Figure 5). As expected, from the small number of clinical samples, we did not obtain statistically significant differences in Rab10 phosphorylation between any of the groups (Figure 5). This is considered further in the discussion. Curiously, donor 22 (G2019S) who displayed unusually low levels of phosphorylated Rab10 (Figure 5C,D) had relatively high levels of LRRK2 Ser935 phosphorylation (Figure 4C).

### Side-by-side comparison of LRRK2-mediated phosphorylation of Rab10 in neutrophils and PBMCs

**Figure 6.**
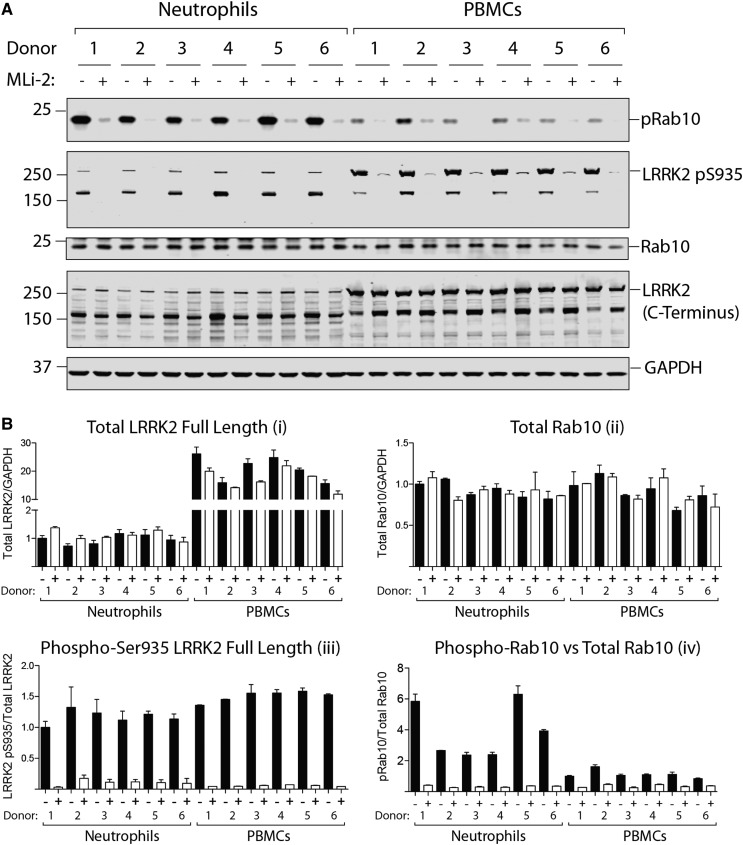
Comparison of LRRK2-mediated phosphorylation of Rab10 in neutrophils and PBMCs. (**A**) Both neutrophils and PBMCs were prepared from the same six healthy donors and treated with or without 100 nM MLi-2 for 30 min. Cells were then lysed and 10 µg of whole cell extract subjected to quantitative immunoblot analysis with the indicated antibodies (all at 1 µg/ml antibody) and the membranes were developed using the Odyssey CLx scan Western Blot imaging system. (**B**) Quantitation of immunoblots was undertaken analysing total LRRK2 (full length)/GAPDH (**Bi**), total Rab10/GAPDH ratio (**Bii**), phospho-Ser935 LRRK2 (full length)/total LRRK2 (full length) (**Biii**) and total phospho-Thr73 Rab10/total Rab10 (**Biv**).

To compare LRRK2-mediated phosphorylation of Rab10 in neutrophils and PBMCs, we isolated neutrophils and PBMCs from the same healthy donors (Figure 6). Flow cytometry was used to assess the proportion of B cells (CD19-positive), monocytes (CD14-positive), T cells (CD3-positive) and contaminating neutrophils (CD66b-positive) in each of the six PBMC preparations (Supplementary Figure S3). This confirmed expected heterogeneity of PBMCs, with levels of monocytes ranging from 6.6 to 15%, B cells from 3.8 to 16%, T cells from 55 to 65% and contaminating neutrophils from 1 to 6% (Supplementary Figure S3).

Immunoblot analysis of PBMCs revealed that full-length LRRK2 was the major species, with only low levels of the ∼170 kDa form observed. Full-length LRRK2 levels were 20- to 30-fold higher in PBMCs compared with neutrophils (Figure 6Bi). Levels of Ser935 phosphorylation normalised to full-length LRRK2 were similar in PBMCs and neutrophils from different donors, with less than 1.5-fold variance between the six donors (Figure 6Biii). Although levels of Rab10 protein were similar in neutrophil and PBMC preparations (Figure 6Bii), 2.5- to 6-fold higher levels of Rab10 Thr73 phosphorylation were observed in neutrophil samples compared with PBMCs (Figure 6Biv). Neutrophils from donor 1, 5 and 6 displayed ∼2-fold higher phosphorylation of Rab10 than other donors, but this increase was not observed in PBMC samples from these donors (Figure 6iv).

### Dose and time dependence of LRRK2 inhibitors on LRRK2 Ser935 and Rab10 phosphorylation in neutrophils

**Figure 7.**
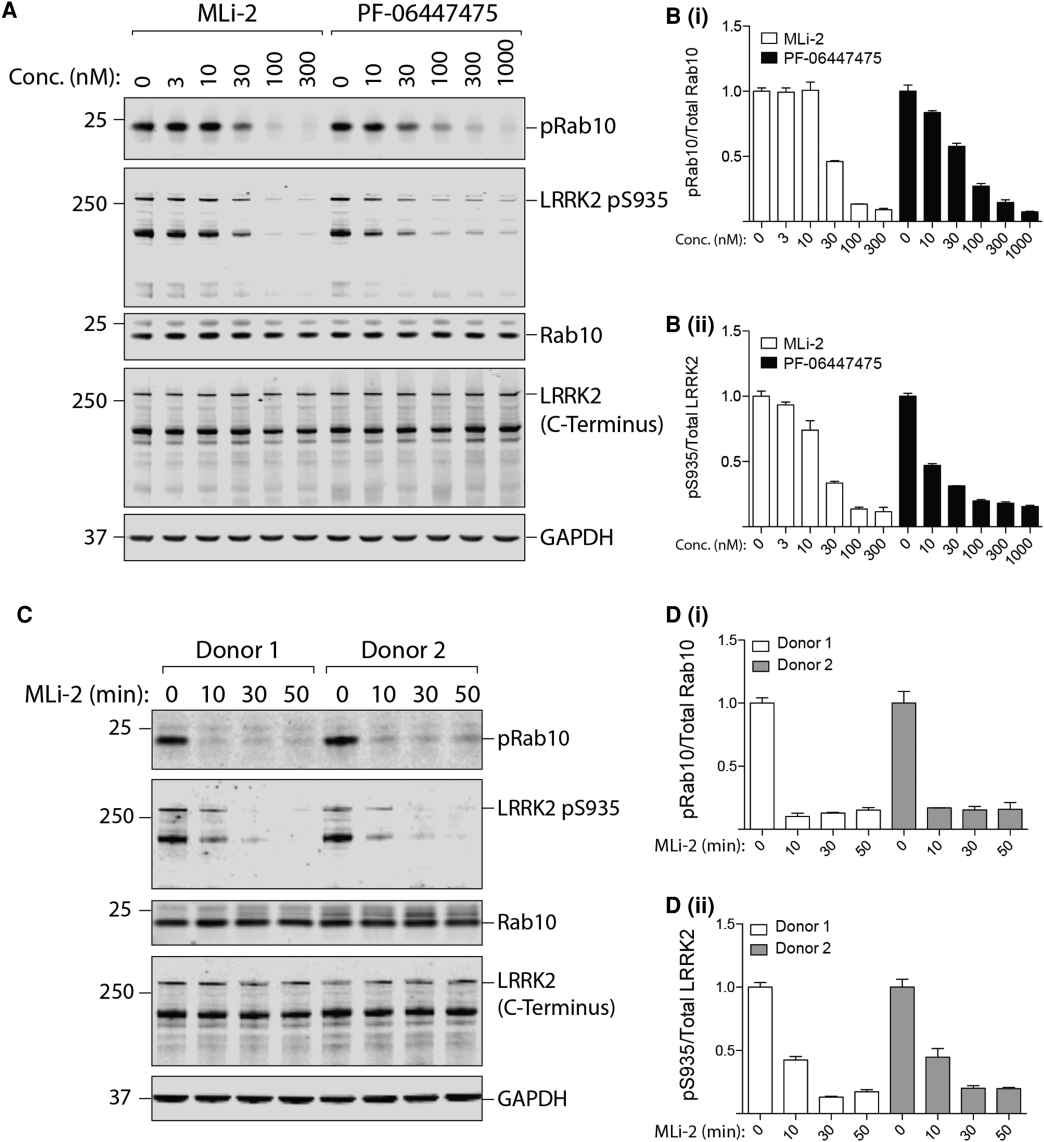
Dose–response curve and time course analysis of LRRK2-mediated Rab 10 phosphorylation in neutrophils. (**A**) Neutrophils were isolated from two healthy donors and treated with the indicated concentrations of MLi-2 or PF-06447475 for 30 min. Cells were then lysed and 10 µg of whole cell extract subjected to quantitative immunoblot analysis with the indicated antibodies (all at 1 µg/ml antibody) and the membranes were developed using the Odyssey CLx scan Western Blot imaging system. (**B**) Quantitation of immunoblots was undertaken by analysing phospho-Thr73 Rab10/total Rab10 ratio (**Bi**) and phospho-Ser935 (full length)/total LRRK2 (full length) ratio (**Bii**). (**C** and **D**) Quantification of immunoblots as above, phospho-Thr73 Rab10/total Rab10 ratio (**Di**) and phospho-Ser935 (full length)/total LRRK2 (full length) ratio (**Dii**), except that neutrophils were isolated from two healthy donors and treated with MLi-2 at concentrations of 100 nM for the indicated times. Similar results were obtained in two independent experiments.

To assess LRRK2 inhibitor dose response, we treated human neutrophils with increasing concentrations of two structurally diverse inhibitors namely MLi-2 [26] and PF-06447475 [36] for 30 min (Figure 7A). With MLi-2, we saw no effect on Rab10 phosphorylation in doses up to 10 nM, a 50% reduction in phosphorylation with a dose of 30 nM and a suppression of Rab10 phosphorylation to near basal levels with doses of 100 nM and above (Figure 7Bi). The dose-dependence curve of the PF-06447475 inhibitor was similar with ∼50% inhibition of Rab10 phosphorylation observed at 30 nM (Figure 7Bi). With regards to Ser935 phosphorylation, both MLi-2 and PF-06447475 inhibited phosphorylation slightly more potently; i.e. moderate inhibition observed at 10 nM and 60–70% inhibition at 30 nM (Figure 7Bii).

We also explored the kinetics of the MLi-2 inhibitor mediated Rab10 Thr73 and LRRK2 Ser935 dephosphorylation in human neutrophils, which revealed that Rab10 was fully dephosphorylated within 10 min following treatment with 100 nM MLi-2 (Figure 7D). Dephosphorylation of LRRK2 Ser935 was slower, with only partial dephosphorylation observed at 10 min and complete dephosphorylation at 30 min of MLi-2 treatment (Figure 7E).

### The importance of including DIFP in the neutrophil lysis buffer

**Figure 8.**
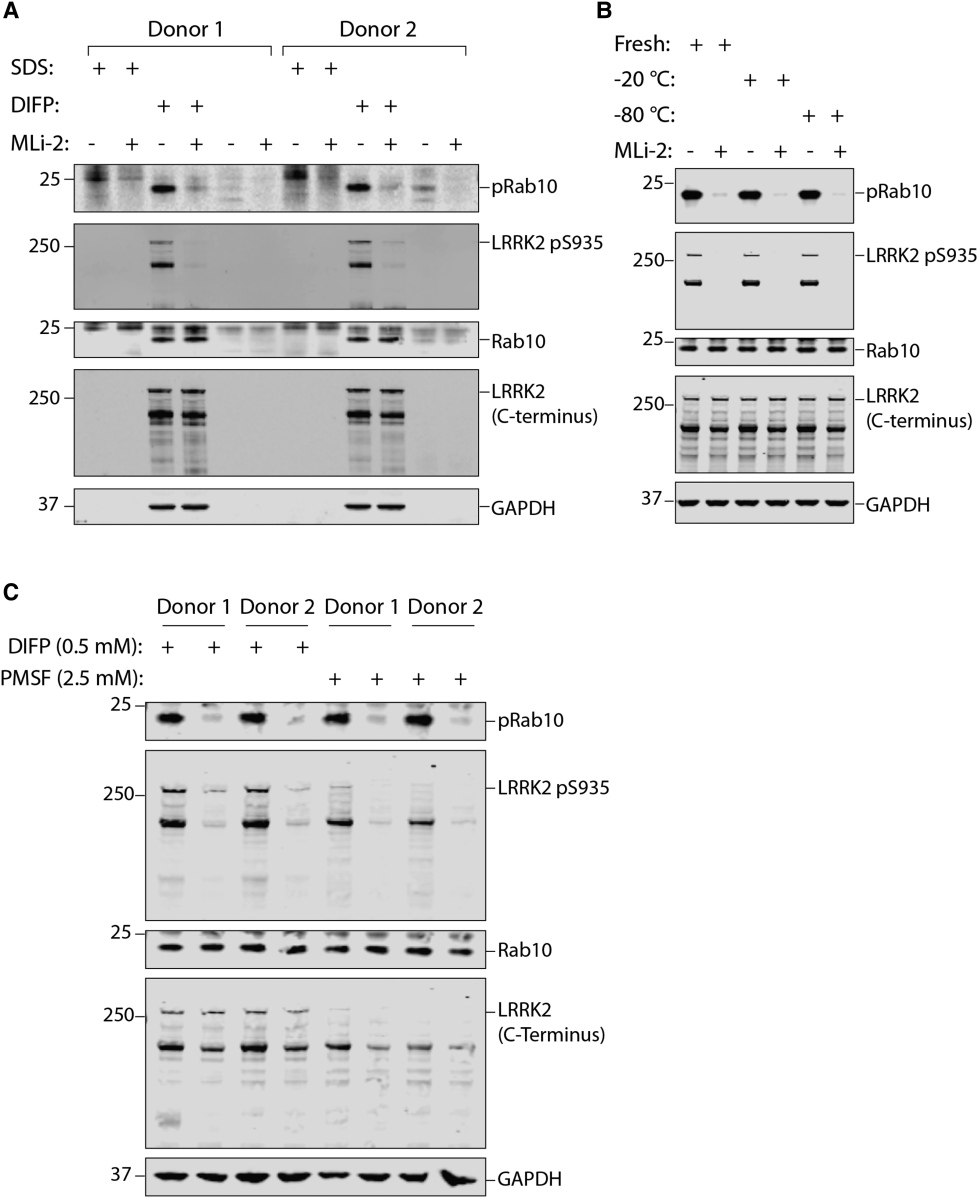
Assessing the importance of DIFP for the prevention of proteolytic degradation in neutrophils. (**A**) Neutrophils were isolated from two healthy donors and treated with 100 nM MLi-2 for 30 min. Cells were lysed in the presence or absence of SDS (1% by volume) and 0.5 mM DIFP as indicated. Whole cell extract (10 µg) subjected to quantitative immunoblot analysis with the indicated antibodies (all at 1 µg/ml antibody) and the membranes were developed using the Odyssey CLx scan Western Blot imaging system. Similar results were obtained in two independent experiments. (**B**) As in (**A**) except that cells were lysed with freshly prepared lysis buffer containing 0.5 mM DIFP or the same buffer that had been prepared 1 week ahead of time and stored at −20 or −80°C as indicated. Similar results were obtained in two independent experiments. (**C**) As in (**A**), except that cells were lysed in the presence of either 0.5 mM (DIFP) or 2.5 mM isopropanol PMSF. DIFP was prepared at 0.5 M in isopropanol and added to the lysis buffer immediately prior to use. PMSF was dissolved at 0.1 M in isopropanol and added to the lysis buffer immediately prior to use.

As the DIFP protease inhibitor is a potent organophosphorus neurotoxin, we investigated whether it was essential to add this to the neutrophil lysis buffer. We therefore lysed human neutrophils with or without DIFP and, in the first instance, explored whether DIFP could be replaced with 1% (w/v) SDS, which has been used to lyse neutrophils in other studies [37]. This strikingly revealed that when DIFP is substituted with 1% (w/v) SDS, LRRK2, Rab10 and even the GAPDH loading control were undetectable, demonstrating the importance of including a highly potent protease inhibitor (Figure 8A). We investigated whether it was possible to store the lysis buffer containing DIFP at −20 or −80°C rather than having to add it to cell lysates immediately prior to use. This revealed that frozen lysis buffer containing DIFP was as effective in preserving LRRK2, Rab10 and GAPDH as the freshly prepared buffer (Figure 8B). Subsequently, we attempted to replace 0.5 mM DIFP with 2.5 mM PMSF, a less potent serine protease inhibitor that is not as toxic as DIFP. We found that Rab10 phosporylation was equally well preserved in PMSF-containing lysis buffer compared to DIFP-containing lysis buffer (Figure 8C). However, the LRRK2 protein, especially the full-length form, was less well preserved with PMSF compared with DIFP (Figure 8C).

### Robust LRRK2-mediated Rab10 phosphorylation up to 24 h after venesection

**Figure 9.**
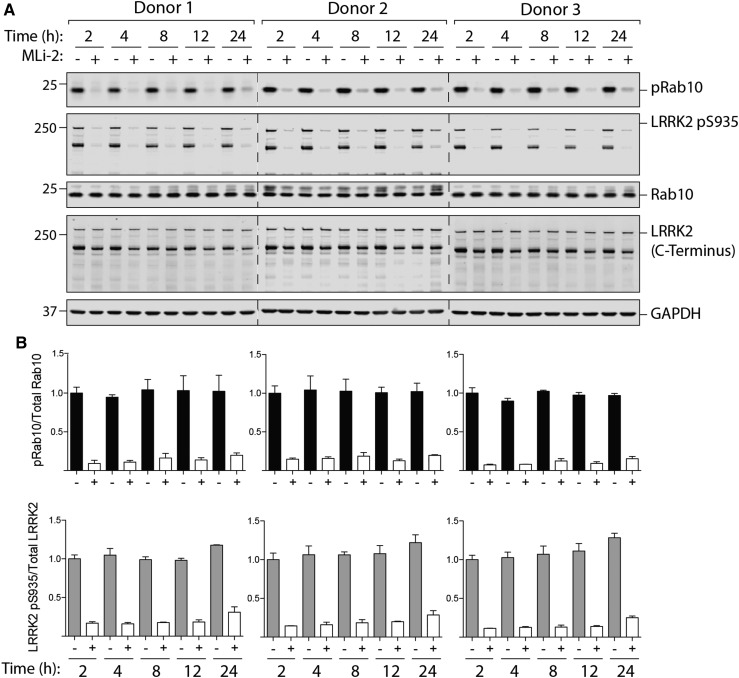
Detection of LRRK2-mediated Rab phosphorylation in neutrophils isolated up to 24 h after venesection. Blood was drawn from three healthy donors and left unprocessed at room temperature. At the indicated times, neutrophils were isolated from the blood and treated with or without MLi-2 at 100 nM for 30 min. (**A**) Neutrophils were then lysed and 10 µg of whole cell extract subjected to quantitative immunoblot analysis with the indicated antibodies (all at 1 µg/ml antibody) and the membranes were developed using the Odyssey CLx scan Western Blot imaging system. Similar results were obtained in two independent experiments. (**B**) Quantitation of immunoblots was undertaken by analysing phospho-Thr73 Rab10/total Rab10 ratio (top panel) and phospho-Ser935 (full length)/total LRRK2 (full length) ratio (bottom panel).

We next explored whether a delay in purifying neutrophils from human blood would affect on LRRK2-dependent Rab10 phosphorylation. Blood was drawn from three healthy donors and left unprocessed at room temperature for up to 24 h before neutrophils were isolated and with or without MLi-2 inhibitor treatment. Immunoblot analysis of these samples reveals that storing blood for up to 24 h does not compromise LRRK2-dependent phosphorylation of Rab10 (Figure 9).

### Monitoring LRRK2-mediated Rab10 phosphorylation in human neutrophils by Phos-tag analysis

**Figure 10.**
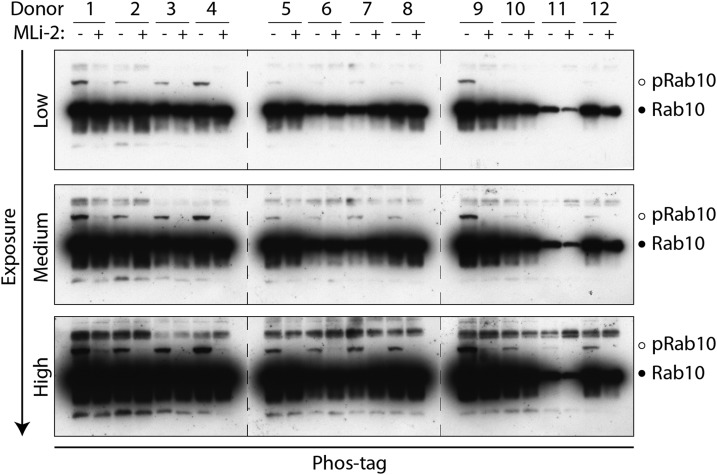
Phosphorylation of endogenous Rab10 in human neutrophils analysed by Phos-tag polyacrylamide electrophoresis. Neutrophils were isolated from 12 healthy donors and treated with or without 100 nM MLi-2 for 30 min. Cells were then lysed and 10 µg of whole cell extract subjected to electrophoresis in which the Phos-tag acrylamide was polymerised into the polyacrylamide gel in order to retard the electrophoretic mobility of LRRK2-phosphorylated Rab10. The gels were subjected to immunoblot analysis with an antibody that specifically recognises Rab10 (1 µg/ml antibody), and the band corresponding to phosphorylated and non-phosphorylated Rab10 is marked with open and filled circles, respectively. A low (top panel), medium (middle panel) and high exposure (bottom panel) of the immunoblot are shown. Similar results were obtained in two separate experiments.

Finally, we investigated whether Rab10 phosphorylation in neutrophils could be monitored by employing the Phos-tag assay, in which the retardation of LRRK2-phosphorylated Rab10 is visualised following Phos-tag polyacrylamide gel electrophoresis [15]. Neutrophils were prepared from 12 healthy volunteers, with or without 100 nM MLi-2 inhibitor treatment and subjected to Phos-tag analysis. As shown in Figure 10, LRRK2-mediated phosphorylation of Rab10 was extremely hard to detect and high exposure of immunoblots was required to observe the LRRK2 phosphorylated species of Rab10 that was sensitive to MLi-2 (Figure 10). Stoichiometry of Rab10 phosphorylation was also exceedingly low (Figure 10).

## Discussion

There is compelling evidence for the role of LRRK2 and in particular its kinase function in Parkinson's disease. This has sparked considerable interest in exploiting LRRK2 as a drug and biomarker target. Here, we describe a facile and robust assay for monitoring LRRK2 kinase pathway activity by measuring its effect on phosphorylation of its physiological substrate Rab10 in (a) the homogenous pool of human peripheral blood neutrophils and (b) with the use of a highly selective phospho-specific Rab10 antibody (MJFF-pRab10 monoclonal antibody).

The rational for using human peripheral blood neutrophils is that they represent a homogenous subset of cells that make up the dominant leukocyte population in humans [38–41]. More importantly, neutrophils express relatively high levels of both LRRK2 and Rab10 [34] (Figure 1). In contrast, the remaining pool of leukocytes, PBMCs, is heterogeneous, and LRRK2 and Rab10 expression is variable. Significant expression of LRRK2 in PBMCs is only found in monocytes [34] (Figure 1) that make up 5–20% of PBMCs. A priori, neutrophils appear to be the most suitable peripheral blood cell type for studying LRRK2 pathway activity. We therefore developed a protocol for peripheral blood neutrophil purification based on negative selection that, within 40 min of venesection, allows isolation of up to 99% pure and viable neutrophils while also yielding relatively high amounts of protein (Figure 2A).

When looking at the total LRRK2 levels in neutrophil lysates, we noticed two bands: one at the expected size for full-length LRRK2 at ∼286 kDa and a shorter one at ∼170 kDa. We suspect that the truncated ∼170 kDa band corresponds to a proteolytic species lacking the N-terminal domain resulting from the high protease activity present in neutrophils, but cannot exclude an alternative splice variant. To our knowledge, no other LRRK2 isoform of that size has been reported [11,42]. The levels of both species of LRRK2 are fairly constant among individual healthy donors (Figure 2Bi,ii), but full-length LRRK2 varied more in the patient samples we analysed (Figure 4A,B). We also observed a moderate reduction in the ∼170 kDa form of LRRK2 after 30 min MLi-2 treatment (Figure 2Bii), but not of the full-length LRRK2 (Figure 2Bi). This suggests that MLi-2 inhibitor binding promotes degradation of the ∼170 kDa form of LRRK2. It has recently been reported that various LRRK2 inhibitors induce destabilisation and degradation of LRRK2, generally over a longer period than 30 min [43,44]. In contrast, a high dose of 1000 nM PF-06447475 did not affect LRRK2 levels, suggesting that this effect will be dependent on the inhibitor used (Figure 7A). With regard to Rab10, expression levels are fairly constant (Figures 2Ci and 4C).

For assessing LRRK2 kinase pathway activity, we have utilised the newly developed phospho-specific MJFF-pRab10 monoclonal antibody that selectively recognises Rab10 phosphorylated by LRRK2 at Thr73 [18]. Unlike Ser935 LRRK2 (see Introduction), the Thr73 Rab10 site is directly phosphorylated by LRRK2 and would be expected to represent a more reliable readout of LRRK2 kinase activity and inhibition thereof. Consistent with the notion that Rab10 phosphorylation represents a more direct and dynamic reflection of LRRK2 activity than Ser935 phosphorylation, we observed higher biological variation in the levels of Rab10 phosphorylation between the 12 healthy donors than with Ser935 phosphorylation (Figure 2Cii,iii). For the purpose of quantifying Rab10 phosphorylation, normalisation can be undertaken using either total Rab10 or GAPDH. It is also possible to calculate the change in phosphorylation of Rab10 observed with and without MLi-2 and then normalise to either total Rab10 or GAPDH, to account for background Rab10 phosphorylation (Figure 5C,D). Until more data is available, we would advocate normalising in all possible ways. In future studies, we would also recommend using 200–300 nM MLi-2 (rather than 100 nM) to ensure that LRRK2-mediated Rab10 phosphorylation is completely reduced, as we observed slightly enhanced suppression of Rab10 phosphorylation at 300 nM MLi-2 compared with 100 nM (Figure 7A,Bi). Finally, the level of Rab10 phosphorylation does not depend on the total amount of Rab10 (Figures 2C and 4D).

The data obtained with the phospho-immunoblotting approach is vastly more robust and sensitive than the Phos-tag method (compare Figure 2 with Figure 10). Comparison of these results highlights the quality and sensitivity of the MJFF-pRab10 monoclonal antibody, which can readily assess low picogram levels of Rab10 phosphorylation in whole cell extracts [18]. We would not recommend the Phos-tag assay for the quantitative assessment of Rab10 phosphorylation in neutrophils, in particular with a view towards clinical translation (compare Figure 2 with Figure 10).

Paramount for the phospho-immunoblot approach is the availability of an antibody that is fully selective against the Rab10 Thr73 phospho-epitope and does not cross-react with other phosphorylated Rab proteins. Another phospho-Rab10 polyclonal antibody has recently been published [17], but its selectivity towards other LRRK2 phosphorylated Rab proteins was not investigated. Based on our experience of generating numerous phospho-polyclonal Rab10 antibodies in sheep and rabbit [18], it is unlikely that any polyclonal phospho-Rab10 antibody would be selective. For future work, we recommend that only antibodies that have been demonstrated to be selective for only a single LRRK2 phosphorylated Rab protein are employed for the quantitative *in vivo* assessment of LRRK2 activity; similar to the characterisation of the MJFF-pRab10 antibodies used in this study [18].

We went on to explore the practicability of using our assay in the clinical setting to test how sample collection, processing, as well as any other uncontrollable and unforeseeable aspects that are intrinsic to clinical practise might affect the outcome. We recruited 13 healthy controls, 7 patients with sporadic Parkinson's, 5 patients with G2019 LRRK2 associated Parkinson's and 1 asymptomatic G2019S LRRK2 carrier (Supplementary Tables S1 and S2). By including participants with LRRK2 G2019S associated Parkinson's, we anticipated to gain more insights into the effect size of LRRK2 kinase activity by means of Rab10 phosphorylation. In a mouse model, a G2019S knock-in mutation in the homozygous state results in an ∼2-fold increase in Rab10 phosphorylation [14,15,18]. One would therefore expect a lower, perhaps ∼1.5-fold increase in Rab10 phosphorylation in the heterozygous state, which is equivalent to a G2019S heterozygous patient. This is a very modest change in LRRK2 activity to measure against the background of normal biological variation in a patient. The detection of a robust change in Rab10 phosphorylation would have required much larger numbers of participants to begin with, if the aim had been to demonstrate a significant difference between the groups. Therefore, the main result is that our assay is robust and can be used in the clinical setting.

We speculate that G2019S LRRK2 mutation carriers would have the highest levels of Rab10 phosphorylation followed by those with sporadic Parkinson's, while healthy controls would have the lowest levels. This trend is observed when LRRK2-mediated Rab10 phosphorylation is normalised to GAPDH (Figure 5A,C). In the G2019S group, there was one patient, donor 22 (Supplementary Table S2), with much lower Rab10 phosphorylation which brings the group's average down (Figure 5). In future work, it would be interesting to include patients with other pathogenic LRRK2 mutations, for example the Basque R1441G LRRK2 mutation. This mutation is of particular relevance in terms of proof-of-principle, as studies in R1441G/C knock-in mouse models have shown to have an at least 2-fold greater effect on LRRK2-mediated Rab10 phosphorylation than the G2019S mutation [14,15,18].

As many other groups have studied LRRK2 in PBMCs [17,45,46] and international Parkinson's bio-repositories collect PBMCs and as far as we are aware of not neutrophils, we undertook a side-by-side comparison of neutrophils and PBMCs. In PBMCs, robust levels of LRRK2 and total Rab10 were observed, with the full-length LRRK2 being the predominant species (Figure 6). From published mass spectrometry data, one would expect that neutrophils display higher LRRK2 activity than PBMCs [34] (Figure 1). Consistent with this, we observe 2.5- to 6-fold higher phosphorylation of Rab10 in neutrophils compared with PBMCs (Figure 6). Although immunoblotting does not reveal higher levels of LRRK2 in neutrophils, this is likely explained by LRRK2 undergoing considerable proteolytic degradation in neutrophil extracts when compared with PBMCs. In our opinion, due to the heterogeneity of LRRK2 expressing cells in PBMCs, PBMCs may be unsuitable for the purpose of monitoring *in vivo* LRRK2 kinase pathway activity with Rab10 phosphorylation as a readout. We would also recommend that Parkinson's bio-repositories consider including neutrophils as an additional bioresource.

The IC_50_ for MLi-2 on Rab10 Thr73 dephosphorylation in neutrophils (30 nM) is higher than what has previously been observed in mouse embryonic fibroblasts (3–10 nM) [15] (Figure 7Bi). This slightly higher IC_50_ value could be explained by the high levels of LRRK2 activity in neutrophils (Figure 1). The IC_50_ for PF-06447475 on Rab10 dephosphorylation has not been reported. Time course analysis reveals that almost complete dephosphorylation of Rab10 Thr73 is achieved within 10 min, while LRRK2 Ser935 dephosphorylation is more protracted. This is similar to what was observed in mouse embryonic fibroblasts [15], suggesting that Rab proteins are dephosphorylated rapidly after inhibition of LRRK2. As at least 30 min are required for maximal dephosphorylation of Ser935, it is important to maintain the LRRK2 inhibitor treatment period at 30 min in future studies (Figure 7D,E). Our work also demonstrates that standard lysis buffer not containing serine protease inhibitors is not sufficient to preserve LRRK2 and Rab10 proteins in neutrophil lysates (Figure 8A). Throughout the present study we have used the highly potent serine protease inhibitor DIFP (0.5 mM), as part of our neutrophil lysis buffer (Figure 8A). To minimise handling and exposure to DIFP, which is highly toxic, we have successfully shown that pre-prepared lysis buffer containing DIFP can be aliquoted and stored at −20°C or −80°C for subsequent use (Figure 8B). We have also substituted DIFP with PMSF (2.5 mM), which is a less potent serine protease inhibitor. At least for monitoring the Rab10 protein as well as its phosphorylation, PMSF works well (Figure 8C). However, we find that PMSF is less efficient at preserving LRRK2. As discussed above, LRRK2 is much more susceptible to proteolysis in particular in neutrophils when compared with other cell types such as PBMCs. In future work, it would be interesting to evaluate other serine protease inhibitors and/or other protein lysis strategies, to explore avenues for improved preservation of the LRRK2 protein in neutrophils.

In summary, we have developed an assay to robustly measure LRRK2 kinase pathway activity by monitoring Rab10 phosphorylation in human peripheral blood neutrophils. Neuroprotective and disease-modifying trials in neurodegenerative conditions have largely failed in the past. There is reasonable hope that this may be different in LRRK2 associated Parkinson's. Orally bioavailable, brain penetrant LRRK2 inhibitors have been generated and individuals who have not yet developed or are early on in their disease course can be genetically stratified based on their LRRK2 genotype. Measuring LRRK2-mediated phosphorylation of Rab10 in neutrophils or other tissues might represent a biomarker that allows for additional patient stratification. We acknowledge that this may be challenging due to the moderate changes of LRRK2 kinase activity associated with pathogenic mutations. Also, our assay captures a peripheral rather than a central readout of LRRK2 kinase function, but to date no non-invasive central measure of LRRK2 activity exists. In the present study, we have highlighted key considerations why neutrophils are a useful biomaterial for analysing LRRK2 pathway activity and the importance of using highly specific phospho-Rab protein antibodies. We also demonstrate the relative ease and feasibility of performing our assay in the clinical setting. We envision that our LRRK2 kinase pathway assay may find wider use for pharmacodynamic and target engagement studies for future LRRK2 inhibitor clinical trials. In future work, it would also be exciting to explore the biological role that the LRRK2 pathway plays in neutrophils and whether it is involved in controlling migration to the sites of the infection and/or phagocytosis and destruction of pathogens.

## Abbreviations

APS: ammonium persulfate
DIFP: diisopropylfluorophosphate
DAPI: 4′,6-diamidino-2-phenylindole
FBS: foetal bovine serum
FC: FCgamma receptors
GAPDH: glyceraldehyde 3-phosphate dehydrogenase
LRRK2: leucine-rich repeat kinase 2
PBMCs: peripheral blood mononuclear cells
PMSF: phenylmethane sulfonyl fluoride
PBS: phosphate-buffered saline
Roc/Cor: Ras of complex/C-terminal of Roc
TEMED: tetramethylethylenediamine
