# Supplementary material for: Interrogating Parkinson's disease LRRK2 kinase pathway activity by assessing Rab10 phosphorylation in human neutrophils
Source: Biochem J. 2018 Jan 2;475(1):23–44. doi: 10.1042/BCJ20170803 (PMC5748842; doi:10.1042/BCJ20170803)
Supplement: Supplementary Figures and Tables [file BCJ-475-23-s1.pdf]

Supplementary Figure 1

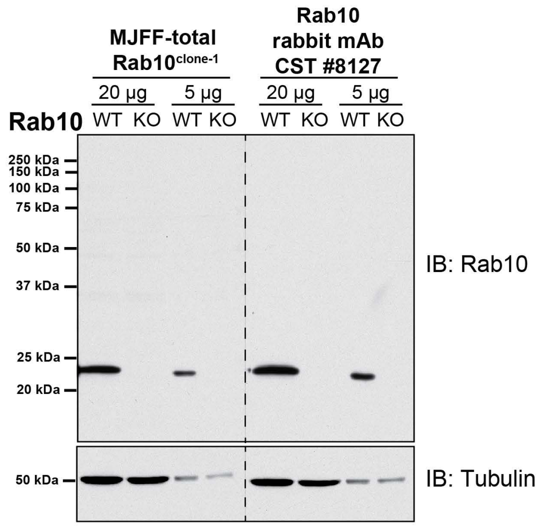

Supplementary Figure 1. **Characterisation of MJFF-total Rab10$^{clone\text{-}1}$ mouse monoclonal antibody**. The MJFF-total Rab10$^{clone\text{-}1}$ mouse monoclonal antibody produced by Nanotool Antibodies was used for immunoblot analysis of the indicated amounts of cell extracts derived from previously described wild type and Rab10 knock-out A549 [15]. For comparison, we undertook a side by side analysis with the best commercial Rab10 antibody that we have been using namely the Cell Signaling Technology (#8127) rabbit monoclonal antibody.

Supplementary Figure 2

Donor 3

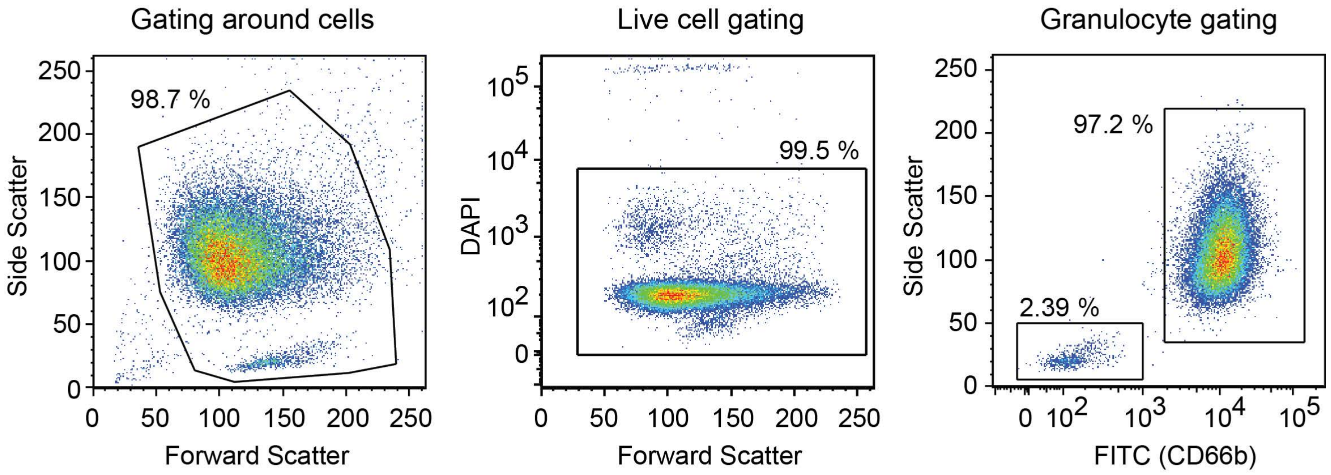

Supplementary Figure 2. **Flow cytometry analysis to determine the purity and viability of isolated neutrophils**. Purified neutrophils from 12 donors were stained with the granulocyte cell surface marker CD66b FITC and the cell viability dye DAPI. Cells were analysed by flow cytometry revealing that isolated cells had a viability >98 % and a purity >96 %. The data shown is from donor 3. Viability and purity data for all twelve donors is provided in Figure 2A.

Supplementary Figure 3

A

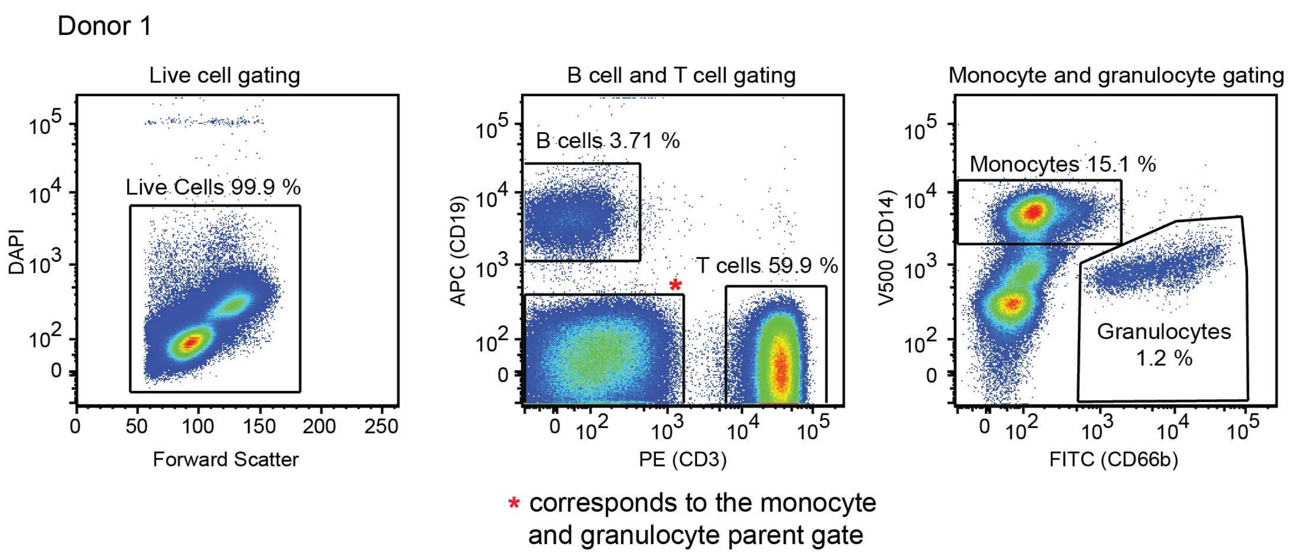

B

Sample	B cells (CD19 positive)	Granulocytes (CD66b positive)	Monocytes (CD14 positive)	T cells (CD3 positive)	Total
Donor 1	3.71	1.2	15.1	59.9	79.91
Donor 2	7.78	5.95	6.56	62.9	83.19
Donor 3	10.9	2.59	9.38	55.1	77.97
Donor 4	8.08	1.01	10.3	65.3	84.69
Donor 5	16.1	4.1	10	55.4	85.6
Donor 6	13	1.83	7.86	58.7	81.39

Supplementary Figure 3. **Flow cytometry analysis to determine the composition of PBMC**s. PBMCs isolated from six donors were stained with CD19 APC (B cell marker), CD3 PE (T cell marker), CD14 V500 (monocyte marker), CD66b FITC (granulocyte marker) and the cell viability dye DAPI. (A) Flow cytometry plots and population gating for donor 1. (B) The proportion of B cells, granulocytes, monocytes and T cells in PBMC isolated from six donors. The data shown is the percentage of each sub-population relative to the total number of cells.

Supplementary Table 1. Overview of demographics of clinical study participants. Overview of demographics and clinical information for the 26 participants. Abbreviations: UPDRS = Unified Parkinson's Disease Rating Scale. NA = not applicable. LDDE = Levodopa daily dosage equivalent. DBS = Deep Brain Stimulation. MoCa = Montreal Cognitive Assessment. Values are median (range). Missing data = MD: ** MD from 4; * MD from 1.

	Control	Sporadic PD	G2019S LRRK2 PD	G2019S LRRK2 Carrier
**Number**	13	7	5	1
**Age (years)**	70 (52;89)	69 (52; 80)	71 (40; 91)	65
**Gender**	8M/5F	3M/4F	1M/4F	1M
**Disease duration (years)**	NA	13 (0; 30)	10 (1; 20)	NA
**UPDRS-III**	NA	35 (21; 48)**	27 (7; 38)	3
**UPDRS-IV**	NA	0**	0	0
**LDDE (mg)**	NA	500 (0; 825)* +2DBS	500 (200; 800) +1DBS	NA
**MoCa**	25 (21; 30)	25 (18; 30)*	25 (12; 30)	30

Supplementary Table 2. Detailed information of clinical study participants. A total of 26 participants were recruited; amongst them 5 patients with G2019S associated Parkinson's, 1 G2019S LRRK2 carrier, 7 patients with sporadic Parkinson's and 13 healthy controls. Abbreviations: UPDRS = Unified Parkinson's Disease Rating Scale. NA = not applicable. LDDE = Levodopa daily dosage (mg). DBS = Deep Brain Stimulation. MoCa = Montreal Cognitive Assessment. Values are median (range). Missing data = MD. TREND = Tuebinger evaluation of risk factors for early detection of neurodegeneration.

	Gender	Ethnicity	Age	Condition	Disease duration	MDS-UPDRS III&IV	MoCa	LDDE
1	female	caucasian	65	LRRK2 G2019S PD	1	7 / 0	27	200
2	male	hispanic	75	Sporadic PD	8	md	terminated after 30min	400
3	female	caucasian	73	Sporadic PD	30	md	md	700 + DBS
4	female	caucasian	67	Sporadic PD	10	36	19	md
5	female	caucasian	52	Control	na	na	md	na
6	female	caucasian	59	Control	na	na	md	na
7	male	arab	40	LRRK2 G2019S PD	4	29 / 0	30	425
8	male	caucasian	80	Sporadic PD	16	48 / 0	18	825
9	female	caucasian	70	Control/TREND	na	na	26	na
10	male	caucasian	76	Control/TREND	na	na	23	na
11	male	caucasian	76	Control/TREND	na	na	25	na
12	male	caucasian	78	Control/TREND	na	na	21	na
13	female	caucasian	71	Control/TREND	na	na	24	na
14	male	caucasian	79	Control/TREND	na	na	24	na
15	female	caucasian	61	Sporadic PD	5	21 / 0	30	200
16	male	caucasian	78	Control/TREND	na	na	22	na
17	male	caucasian	68	Control/TREND	na	na	26	na
18	male	caucasian	89	Control	na	na	md	na
19	female	caucasian	86	LRRK2 G2019S PD	16	na	12	675
20	male	caucasian	65	LRRK2 G2019S carrier	na	3	30	Na
21	female	caucasian	66	Control	na	na	30	na
22	female	caucasian	91	LRRK2 G2019S PD	9	38 / 0	28	800
23	male	caucasian	68	Control/TREND	na	na	27	Na
24	female	caucasian	73	Sporadic PD	25	md	29	350 + DBS
25	male	caucasian	52	Sporadic PD	1	md	30	None
26	female	caucasian	71	LRRK2 G2019S PD	20	36	26	400 + DBS
